# Insurance market development and economic growth in Africa: Contingencies and thresholds of structural transformation

**DOI:** 10.1016/j.heliyon.2024.e40046

**Published:** 2024-11-01

**Authors:** Sylvester Senyo Horvey, Jones Odei-Mensah, Andre P. Liebenberg

**Affiliations:** a Wits Business School, University of the Witwatersrand, Johannesburg, South Africa; b Department of Finance, School of Business Administration, University of Mississippi, United States

**Keywords:** Economic growth, Insurance, Structural transformation

## Abstract

Despite insurance contributions to economic growth, the intervening role of structural transformation on the insurance-growth nexus remains scarce, leaving a gap in the literature. Motivated by the 2030 Sustainable Development Agenda, which envisages higher structural transformation, financial development and economic progress, this study examines the threshold effects and the joint impact of insurance and structural transformation on economic growth in Africa. The study employed the generalised method of moments and dynamic panel threshold estimations on 38 countries covering 2008 to 2020. The following findings emerge: First, insurance and structural transformation promote economic growth. Second, the results indicate that structural transformation enhances the impact of insurance on economic growth. This suggests that structural transformation matters in driving insurance market development, ultimately promoting economic growth. Third, the empirical evidence further reveals nonlinearities in the relationship and presents that a high level of insurance penetration stimulates growth and vice versa. More so, the synergistic effect of insurance and structural transformation on economic growth is more substantial at higher threshold levels. This implies that though insurance spurs economic growth, its impact is more effective through the intervening role of a strong structural transformation. Therefore, this study calls for government intervention and policies to improve structural transformations and form insurance synergies to promote growth.

## 1 Introduction

The performance of the insurance industry has been a feature of the growth path of developed and developing economies. Insurers pool risk together, provide employment, promote financial stability to households and safeguard organisational growth and development, thereby contributing to the overall economic growth [1]. By pooling risks together, insurance reduces financial uncertainty, promotes investment and supports trade, commerce and entrepreneurial activity. Insurance also offers financial protection against natural disasters and health issues and is crucial for managing business risks. By insuring these risks, insurance supports innovation and business development, which plays a pivotal role in stimulating economic growth [2]. Besides risk pooling, insurers provide risk management services to businesses to minimise the possibility of adverse events. These services reduce uncertainties and promote growth and sustainability, which in turn contribute to the growth of economies. More so, insurers operate as a business and provide employment opportunities [3]. In this regard, insurers pay salaries, taxes, and dividends to shareholders, all of which offer economic benefits both directly and indirectly. Another avenue through which insurers support the economy is claims settlement given to individuals and companies during adverse events [3]. This protects their income and assets, which can subsequently be used fully or partially for economic development or investment [4]. As a result, companies can continue to operate without fear, given that they will be indemnified if the insured loss occurs. This ensures that their income is not affected; hence, they can provide products and services to the economy, thereby promoting economic growth.

Furthermore, the insurance industry’s ability to adjust and respond to the changing demands of an economy underscores its crucial function in promoting sustainable growth [5]. Thus, a strong insurance industry is more important as countries grow and diversify, emphasising its vital role in the general well-being and success of the economy [6]. Despite its importance, the insurance market in Africa suffers greatly. In 2020, the Swiss-Re Institute explained that the insurance industry in Africa remains the least penetrated globally, contributing about $60,190 million, equivalent to 2.0 % of GDP, far below the global insurance penetration rate of 7 %. Also, Asongu and Odhiambo [7] highlight that only 5 % of Africans have access to insurance. This underscores a considerable gap in insurance protection in Africa, revealing a vast, untapped market brimming with substantial opportunities for growth and development. Given this, insurers must explore innovative ways and leverage evolving structural changes to capitalise on the resurgent economy and opportunities available to spur their growth potential.

A report by PricewaterhouseCoopers (PwC) explains that insurers in Africa should pay keen attention to structural transformational effects, technology and other regulations to enhance their impact on the continent [8]. The importance of structural transformation is further highlighted in the 2030 Sustainable Development Goal (SDG), particularly SDG 9, which emphasises the transition from low to high productivity. This is critical for economic and financial development, encompassing sectors such as the insurance industry [9,10]. This suggests that structural transformation’s impact on the insurance industry must be highlighted because, to accelerate growth, one must first examine the factors that facilitate the growth process. This can be achieved through increased productivity, diversification, job creation and financial deepening. Structural transformation denotes the progressive and continuous reallocation of labour and other productive resources across economic activity, particularly from low to high productivity [11]. This includes rapid urbanisation, a rise in the industrial, manufacturing and service sectors, and demographic changes. This transition from low to high productivity affects every sector of the economy. Geda et al. [12] support this claim by narrating that structural transformation promotes industrial capabilities and enhances the growth of the financial sector, including the insurance industry. Structural transformation develops the insurance market through technological advancements, improved distribution channels, cost reduction, tailored products, and financial literacy, fostering trust and understanding of insurance products [13]. This results in different insurance distribution channels, boosting the insurance market. More so, as countries undergo structural changes, the demand for various insurance products rises due to income growth, urbanisation, increased risk and the formalisation of economic activity. Further, improved industrial and service sectors make robust risk management plans necessary, emphasising the requirement for all-encompassing insurance protection [14]. Additionally, improvements in the financial structures and regulatory frameworks triggered by the structural change also facilitate more trust and activity in the insurance markets.

Against these backdrops, the question remains: how does structural transformation bolster the insurance market in fostering economic growth? The relationship between insurance and economic growth has received some recognition from the literature. However, the results remain inconclusive [6,7,15], highlighting a significant gap in the literature. Some studies argue that insurance fosters economic growth, which is affirmed by its positive results [5,16]. Also, Balcilar et al. [17] investigate the dynamic relationships between insurance and economic growth, revealing short-run and long-run effects. On the other hand, Zouhaier [18] discovered an inverse relationship and attributed this direction to moral and morale hazard problems from the insured. Other scholars have also investigated the bi-directional relationship between insurance and economic growth [19,20] and found mixed, unidirectional and bidirectional relationships. Asongu and Odhiambo [21] explored the threshold dynamics of insurance penetration on economic growth, concluding that insurance enhances growth when it exceeds the minimum critical mass level. A more recent study by Horvey et al. [4] explored the nexus between insurance and inclusive growth, narrating that insurance not only promotes economic growth but also fosters inclusiveness. The authors add that a high level of insurance penetration is essential to achieve inclusive growth in Africa.

Despite these efforts, it can be seen that studies on the insurance-growth nexus have produced divergent results, indicating that the argument between insurance and economic growth is far from settled. As Horvey and Odei-Mensah [5] highlight, the divergence of these results can be related to several factors, including the varying stages of the insurance market and some contingency factors. This aligns with Outreville’s [22] argument, which further suggests the need to explore the conditions required to enable the growth of the insurance markets. As a result, the current debate in the literature increasingly seeks to understand the factors that could potentially induce the performance of the insurance industry in affecting growth. Given this, scholars have begun to explore the intervening role of factors such as financial inclusion [23], terrorism [24]), institutional quality [16] and ICT diffusion [5] on the relationship between insurance and economic growth. In spite of these attempts, scholars have yet to explore the intervening role of structural transformation on the insurance-growth nexus. This oversight presents unresolved issues of how the ongoing structural transformation within economies stimulates the insurance market in affecting economic growth, a gap this study addresses.

Exploring the intervening role of structural transformation on the insurance-growth nexus is critical, given that the growth of insurance companies is largely dependent on the institutional and structural environment. This is affirmed by Horvey et al. [4], who assert that the effectiveness of insurance in driving economic growth varies due to differences in a country’s environmental and economic situation. According to PwC [8], demographic and social changes, rapid urbanisation, sectoral developments and industrialisation, and changes in economic structures present new opportunities for driving the insurance market. Thus, as economies transform, the demand and impact of insurance change, potentially altering its influence on the economy. The African Insurance Organisation (AIO) affirms the importance of structural transformation towards developing the insurance market, positing that these two factors are essential in ensuring the coordination of policy developments to achieve a competitive industry in Africa, thereby stimulating economic growth [25]. Despite the importance of these factors, there remains a gap in the literature on the plausible joint effect of insurance penetration and structural transformation on economic growth, which underscores the need for further exploration. Additionally, the literature suggests that a well-developed insurance market and structural changes are essential to foster economic growth [4,21]. However, it remains unclear at what threshold these factors might propel or weaken economic growth. Addressing this gap is very important to reconcile the divergent results and to develop relevant policies to drive the insurance market, ultimately affecting economic growth.

Given the above, the novelty and significance of the study lie in answering the following three questions: First, what is the impact of insurance and structural transformation on economic growth? Second, how does structural transformation moderate the insurance-growth nexus? Given that previous studies failed to examine the interaction between insurance penetration and structural transformation on economic growth, this study, to the best of our knowledge, is the first to understand this relationship and its effect on economic growth. This provides deeper insights into how the insurance industry responds to the changes in an economy’s structural system to develop its market and foster economic growth. Third, does this study, besides the interactive effects, reveal non-linearities in the relationship? This provides a basis to explore the threshold at which insurance and structural transformation weaken/enhance economic growth. Addressing this issue is essential to understand whether the development of the insurance market and growth in structural reforms are critical conduits for economic growth. By answering these questions, the current study expands the literature by initiating a new discussion on the importance of structural transformation in developing the insurance market, thereby affecting growth, which, to the best of our knowledge, has not yet been explored.

The insurance industry in Africa provides a compelling context and has several policy implications. The African insurance industry is mostly characterised by low penetration and remains the lowest globally, which presents several growth opportunities [4]. Also, due to institutional, historical, and policy considerations, structural transformation in Africa has taken different forms and progressed at different rates in various countries [26]. These variations significantly influence the development of the insurance market and its impact on economic growth. Hence, investigating the intervening role of structural transformation on the insurance-growth nexus is essential. This study provides insights into how structural changes, like the shift from an agriculture to an industrial and service-based economy, bolster insurance’s role in the economy’s progress. This will provide information to countries that want to enhance economic growth by improving the insurance industry through structural transformation. Understanding the relationship can facilitate policy measures customised to optimise the insurance industry’s influence in diverse economic contexts, promoting more equitable and sustainable economic growth. This helps address the low penetration issues in Africa. Also, this study highlights the effect of insurance penetration and structural transformation on economic growth at different threshold levels. This explains how these factors behave at low and high levels, providing information to policymakers on the structural reforms that must be addressed to develop the insurance market. The result further provides governments and regulatory authorities with reforms and policies to sustain and deepen the insurance market. A vibrant and well-developed insurance industry will help stimulate sustainable economic growth among African countries. By presenting perspectives from a region that is mostly underrepresented in studies of global economies, this research adds to the body of knowledge on the insurance-growth literature and provides guidance for other emerging areas dealing with similar structural issues.

Presaging the key findings, the empirical results show that insurance and structural transformation exhibit an enhancing effect on economic growth. Additionally, the significant positive interaction effect suggests that structural transformation propels the impact of insurance in stimulating economic growth. Furthermore, evidence from the dynamic panel threshold regression presents that the interaction between insurance penetration and structural transformation promotes economic growth at higher threshold levels. Below the threshold level, structural transformation exhibits a weakening effect on insurance penetration, affecting growth. This implies that insurance exacerbates economic growth through the intervening role of a robust structural transformation system. The rest of the paper is structured as follows. The next section discusses the literature. Section three outlines the data and methods used for the study. Section four presents the findings and discussion of the results, and conclusions and recommendations are provided in the final section.

## 2 Literature review

### 2.1 Theoretical background

Several theories, including the theory of financial intermediation, the supply-leading hypothesis, and the institutional and economic transformation theories, explain the relationship between insurance, structural transformation and economic growth. The theory of financial intermediation posits that the development and deepening of financial markets, such as the insurance sector, is essential for value creation for economies by channelling resources from savers to borrowers and managing risks [27]. This process distributes resources more effectively, fostering economic growth and development [28]. By allocating resources toward profitable ventures and consolidating risks, insurance serves as a financial intermediary and a shock absorber. This intermediation improves financial stability and fosters economic growth. Haiss and Sümegi [29] expand further by narrating that the insurance industry contributes to the growth of economies through two main avenues: risk transfer and investment. In terms of risk transfer, insurers bear the risk of individuals and other economic agents, enabling them to engage in activities with less financial uncertainty. This provides a safety net to organisations, which aids them to engage in new or expanding projects. By bearing the risks, insurance encourages savings, investment and consumption. This ensures growth and stability in their earnings and reduces volatility, thereby promoting economic growth [30]. Regarding investment, insurance facilitates capital formation by directing premiums toward long-term investments, including investment in various assets, which helps deepen the capital markets and expand the investment range and volumes. This provides a channel through which insurers can engage in infrastructural and economic projects, promoting economic growth.

The above argument aligns with the supply-leading hypothesis, which explains that the development of the financial markets fosters economic growth due to their ability to mobilise savings and investment [20]. This theory postulates that when resources are redistributed from inefficient industries to productive ones, a stable financial system - including the insurance industry - encourages the growth of economies [31]. Therefore, in a setting where natural resources are abundant, institutional frameworks are being established, infrastructure development is increasing, and the population is youthful, insurance may be a game-changer in fostering growth [32]. Horvey et al. [4] add that the micro-level operations of the insurance market offer protection and stability to individuals and companies, while the macro-level financial markets utilise the cash collected from insurance premiums and their activities have ripple effects on other financial markets. This highlights how crucial insurance-related activities are to other financial sectors and the economy. By investing in a range of assets and collecting premiums, insurers provide capital to the economy, promoting the growth of businesses and economies [33].

In addition, scholars argue that the link between insurance and economic growth may vary depending on the stage of an economy’s structural system [5,8]. This can be explained by the institutional development and economic transformation theories. These theories explain how technological innovations, resource reallocation, and sectoral changes transform economies into more diversified and productive economies. These structural reforms lead to the development of organisations, including the insurance markets [34,35]. As economies undergo structural changes, their risk profiles change, increasing the demand for specific insurance products to provide coverage for risks and new businesses. As a result, the transformation in the legal, regulatory, technological and structural systems influences the insurance market, ultimately affecting growth. In the early stages of structural reforms, insurance may have less impact due to undeveloped systems and institutions and vice versa [26]. These theoretical perspectives provide an interesting framework to explore the direct, synergies and threshold dynamics between insurance, structural transformation and economic growth.

### 2.2 Review of insurance and economic growth

The insurance industry is prominently featured in the debate on growth and development in Africa. It serves as a shock absorber, ensures organisational sustainability, and influences the growth path of every economy [3]. Hence, a strong and well-developed insurance industry can boost economic growth significantly [36]. Empirical studies have provided an inconsistent but generally positive relationship between insurance and economic growth. For instance, Horvey and Odei-Mensah [5] reveal a significant positive relationship between insurance, including life and non-life penetration and economic growth. This suggests that the promotion of insurance leads to growth in economies. They further narrate that non-life insurance encourages capital allocation, and life insurance promotes long-term investments, which are essential in spurring the growth of economies. Similarly, Apergis and Poufinas [37] discovered a significant positive relationship between the coefficient of insurance penetration and economic development before and during the global financial crisis (2007–2008). The results remain the same for the non-life and life sectors. This shows that a well-developed insurance market fosters economic growth. This is due to the financial security provided by insurers to individuals and businesses, which promotes growth and investment, leading to overall economic performance [38]. Asongu and Odhiambo [21] support this argument and narrate that to promote economic growth, the insurance penetration for non-life (life) should have a penetration rate of at least 1.81 % (4.15 %), particularly for African countries. Dawd and Benlagha [6] affirm this by indicating that insurance development is pivotal to the growth of economies as it promotes capital accumulation and investment.

Ali [39] found a similar result and explains that insurance drives the growth of economies by creating a capital base and financial security for businesses. Balcilar et al. [17] provide evidence to support this assertion by describing how risk pooling, financial intermediation, loss indemnity, saving mobilisation, and investment possibilities enhance the performance of the insurance market, thereby promoting economic development. Further, Alhassan [20] narrate that the nonlife insurance industry exhibits a significant impact on growth in countries such as Kenya, Gabon, Madagascar, Algeria, Morocco and Nigeria, while the impact of life insurance on growth is more substantial in countries such as Mauritius and South Africa. This could be attributed to the fact that these countries’ life insurance market is stronger. Lee et al. [40] explored a sample of 123 countries and found a causal link between insurance and the growth of economies. However, the relationship varies throughout countries due to differences in income levels and contextual factors. They further narrate that one of the factors likely to drive insurance is the performance of investment in the industry.

Despite the various positive assertions by these scholars, other studies argue that insurance adversely influences economic growth. One such study is Lee et al. [15], who discovered a negative relationship between insurance and growth, suggesting that insurance operations hinder economic growth. They associate this negative relationship with poor institutional climate and structures. The authors explain that in environments characterised by weak governance, poor regulations and high levels of corruption, the insurance sector may struggle with inefficiencies, lack of transparency and market distortions. This makes the industry less competitive, experiences high operational costs and creates several inefficiencies, hindering insurers’ potential to promote economic growth. Similarly, Fernando et al. [41] found an inverse relationship, indicating that a generally poor institutional environment might prevent the insurance industries from growing. They state that when the institutional environment is strong, all sectors, including the insurance market, will be developed.

Some scholars have also examined the bidirectional relationship between insurance and economic growth. Mitrasavic et al. [42] found a two-way relationship between insurance and growth, which suggests that insurance not only stimulates economic growth but that the growth of economies provides avenues for insurance development by increasing the demand for insurance products and services. In the case of Şenol et al. [43], a one-way causal relationship was found between non-life insurance and economic growth, while a two-way relationship exists between life insurance and economic growth. Their findings show that the life insurance industry contributes more to economic growth and has a consistent and long-term impact than the non-life insurance industry. In the case of Zulfiqar et al. [38], a bidirectional relationship was found to exist between non-life insurance and growth. Similarly, Singhal et al. [44] also found a bi-directional relationship for both advanced and emerging economies, suggesting that policymakers promote activities that drive the insurance market development, which will ultimately influence the growth of economies. The causal relationship supports the ‘supply-leading’ and ‘demand-following’ hypothesis, which explains that insurance promotes economic growth and vice versa. According to Scharner et al. [45], as economies get wealthier, households receive higher incomes and can pay a larger percentage of their income on insurance.

Another strand from the literature has also explored the short-run and long-run dynamics of the insurance-growth relationship. Evidence for both short- and long-term effects was discovered by Balcilar et al. [17]. This suggests that insurance has an immediate and long-lasting impact on growth. However, Okonkwo and Eche [46] discovered a unidirectional association among the BRICS economies. They further narrate that the relationship is specific to individual countries and is influenced by several institutional and financial variables. Pradhan et al. [3] also found that insurance presents a long-term impact on the growth of economies, and they argue that long-term sustainable policies are required to enhance the performance of insurance in driving growth. Chukari et al. [47] discovered a long-term positive association between non-life insurance premiums and growth. On the other hand, there is a negative short-run association between non-life insurance and growth. They conclude that the non-life contributes more to growth than the life industry due to their ability to generate capital to safeguard infrastructure, real estate, and capital markets, which helps stimulate economic growth. In the case of Tran and Huynh [48], a unidirectional causality and positive effects between life insurance and growth both in the short and long run were found to exist, supporting the supply-leading hypothesis. In the long run, they find that the non-life industry exhibits a strong impact. This is consistent with Cheng and Hou’s [49] finding, which further argues that life insurance is a panacea for the finance-growth relationship because it absorbs the negative effect of private lending on real economic growth while simultaneously assisting in reducing long-term real growth volatility.

Given the conflicting argument on the relationship between insurance and economic growth, some scholars argue that the performance of the insurance industry will be substantial in the presence of strong environmental conditions. For instance, Asongu and Odhiambo [7] contend that the performance of the insurance industry is contingent on the level of financial development and institutions. The authors present that institutional factors such as control of corruption, regulatory quality, government effectiveness, the rule of law, voice and accountability and political stability are essential to the performance of the insurance industry. This is consistent with Lee et al. [15], who posits that strong institutions provide the proper framework for the effective operation of the insurance business. Effective regulation ensures market stability, consumer protection and insurance solvency. Additionally, Alam et al. [24] explored the role of terrorism in the relationship between insurance and growth and found that terrorism has a detrimental effect on the positive impact of insurance on growth in the Middle East and North American countries. This suggests the need for stability in the political climate for MENA countries for insurance to thrive. Given the importance of contextual factors, Horvey and Odei-Mensah [5] also examined the intervening role of Information and Communication Technology (ICT) on the insurance-growth nexus and affirmed that the impact of insurance on growth is contingent on ICT diffusion. The authors narrate that ICT streamlines insurance operations through automation, digitisation, introduction of new products, and expansion of their customer base. This reduces transaction costs and improves their effectiveness, ensuring the industry’s growth and development. This will ultimately influence the economy. As a result, insurers must employ technology and leverage ICT advancements to enhance their performance and operations.

In summary, the above discussion offers an intriguing perspective on the state of knowledge in the literature. The majority of the existing literature focuses on the direct relationship, revealing divergent arguments between insurance and economic growth. The literature indicates that the impact of insurance differs across countries in terms of income levels, type of insurance, and level of development. Given these contradictory findings, some studies have begun to explore the conditions in which the insurance industry is likely to thrive. However, empirical efforts are insufficient, offering limited perspective on the intervening factors influencing the relationship between insurance and economic growth. This suggests the need for further investigation into other factors likely to affect the insurance-growth relationship. One of the factors that has been ignored is structural transformation, which is essential in the African context, given the continuous transformation of African economies. This is further discussed in the following subsection.

### 2.3 Review of the role of structural transformation on the insurance-growth nexus

Many African countries have envisaged reaching middle-income status in the coming decades. To realise this vision, significant changes in the structural transformation system are required [26]. Despite countries’ efforts to promote transformation, the structural transformation process in Africa has remained subtle [50]. Some reasons include weak institutional frameworks such as weak governance, corruption, and poor legal and regulatory systems [51]. There is also insufficient infrastructure and limited access to finance for productive purposes. These stifle investment, innovation and financial development [52]. When the situation remains unchanged, it may have a detrimental effect on the sectoral development of Africa. Page [53] posits that a lack of access to high-quality education and training programs has resulted in an enormous skills gap in the labour force. The scarcity of skilled labour hampers the adoption of innovative technology and the development of high-value industries. Unlike Western countries, African countries have been behind in successfully transforming their economies and thus upgrading their industrial capabilities to move up the value chain in the last six decades [12]. Notwithstanding, some African countries are redoubling their efforts to industrialise and structurally transform their economies. Post-independence African political leaders are eager, both in intent and in practice, to embark on structural transformation, which is commonly associated with the development of the industrial and service sectors [12]. Although the path of structural transformation differs among African countries, it has been discovered to be an essential feature of industrial and economic development. Hence, African countries could leverage structural transformation to enhance the competitiveness of the insurance industry, which is likely to promote growth. This argument is supported by Chang and Lee [54], who point out that structural change presents several benefits to the insurance industry by opening up a wider variety of economic endeavours to use insurance for investment and risk management. Insurance helps companies handle risks unique to their industry, which in turn promotes the growth of more capital-intensive enterprises and the economy.

According to Davies and Green [55], the insurance industry promotes growth through several mechanisms, including improved financial stability, trade and entrepreneurship, and structural transformations. An improved structural transformation enhances data model and analytics sophistication for better risk identification and quantification. This provides more accurate information for insurers to assess and insure more risks, leading to higher penetration. This will bring about new and innovative methods of distribution of insurance products due to innovative technologies. This suggests that structural change improves the financial benefits of insurance services and creates an atmosphere more conducive to their use [41]. According to Africa Re [56], many African countries’ capacity and capability to respond quickly to natural disasters and strengthen their resilience over time are limited by structural factors. This is affirmed by Horvey and Odei-Mensah [5], who highlight that structural inefficiencies can dampen the development of the insurance market and its growth-lubricating effect. They further claim that in countries with advanced structural transformation, insurance services may be able to help the industrial and service sectors more successfully. However, in economies that have not seen as much change, the presence of informal and subsistence enterprises may restrict insurance benefits. Kedir et al. [57] suggest that the continent will require significant funding to achieve the desired transformation agenda. Government can contribute to this by ensuring improved institutions and structural transformation systems.

Taking the four sectoral value additions (i.e., agriculture, manufacturing, industrial, and service) into consideration, the literature suggests that these individual proxies of structural transformation are critical to the growth and development of the insurance industry. For instance, agriculture remains the most important industry in many developing countries. It still contributes significantly to GDP and is the primary source of employment. This sector in developing economies is characterised by low-income levels, low capital-labour ratios, and the general instability of agricultural production [58]. Agricultural insurance is a more effective and efficient institutionalised mechanism for dealing with the problem. It facilitates the coordination of relief efforts and reduces the direct and indirect costs to the national economy. Li and Wang [59] argue that policy-based agriculture insurance might heighten farmers' interest in participating in insurance by providing premium subsidies. Also, the manufacturing and industrial sectors are facing significant risks in today’s world. Since the manufacturing industry is capital-intensive with an array of operational risks, there is a considerable need for various insurance services. Banker et al. [60] highlight that manufacturing companies require broad insurance coverage as they expand to lower the risks of equipment breakdowns, supply chain disruptions and production delays.

Similarly, the industrial sector requires capital-intensive investment and involves several operational risks [61]. As a result, they rely on insurance to cover most of their operations, including casualty insurance, fire insurance, and risk management services [14,62]. More so, regulatory frameworks of the manufacturing and industrial sectors sometimes impose obligatory insurance needs, which can greatly increase insurance penetration rates. This demand extends beyond property and casualty insurance, including specialised coverages like business interruption and liability insurance. This demand for insurance coverage promotes insurance performance, thereby enhancing economic growth. This is affirmed by Fernando et al. [41], who suggest that industrialisation raises the need for a range of insurance products, including commercial, property, and health insurance, which boosts the industry’s development potential. This implies that the insurance industry becomes increasingly essential to sustaining economic growth as economies move toward more industrial and service-oriented activity. Additionally, the service sector encompasses many businesses, including financial services, education, travel, medical care and professional services. These sectors have unique insurance needs and risks. Sare et al. [63] demonstrate that service-oriented businesses usually require specialised insurance coverages, including cyber insurance, professional liability coverage and insurance against malpractices to reduce operational risks. These insurance requirements are mostly driven by contractual obligations, legal requirements, and the need to safeguard against financial losses resulting from lawsuits and business disruptions. Given this, the expansion of the service sector is likely to contribute to developing the insurance market, which will ultimately improve the growth of economies.

Regarding technological developments, the understanding of digital technology in Africa is evolving and destined to change how insurance operates. Technology enables new ways of doing business in the insurance industry. Pradhan et al. [3] narrate that increased accessibility to smartphones, the internet, the Internet of Things, sensor technologies and satellite communications provides the insurance industry with more data and makes them more informative in their decisions and policy offerings. Technological developments empower insurers to better monitor customer behaviour and risks and design appropriate customised solutions [5]. It provides a digital platform for selling insurance products, pricing and risk selection, anticipating trends, offering risk management services, and changing the underwriting cycle, all of which promote the development of the insurance market [64]. Benlagha and Hemrit [65] argue that ICT is an indispensable tool for accelerating global financial growth. This provides opportunities for insurers to remain connected with policyholders by receiving and responding to their demands through digital devices. This also provides avenues for insurers to operate globally since clients do not need to be physically present to purchase insurance products. Bayar et al. [13] add that ICT leads to improvements in product design by making it possible to provide customised services and products in place of conventional ones, improving risk assessment, and ultimately resulting in more appropriate insurance premiums, leading to the development of economies. Technology also enhances claim turnaround by providing insurance efficiency. Nevertheless, the size and quality of internet infrastructures in Africa pose a significant challenge to advancing insurance development [66].

In terms of infant mortality, the literature reveals that countries with low infant mortality are more responsive to health insurance than those with high infant mortality [67]. This is an important indicator of health because of its sensitivity to structural factors such as socio-economic development and basic living conditions. A lower infant mortality rate is frequently associated with better healthcare service and infrastructure levels [68]. Generally speaking, these nations have highly developed healthcare systems with hospitals, clinics, and qualified medical personnel. In situations like these, insurance is essential because it guarantees that patients may obtain high-quality medical care without facing financial obstacles. In order to cover the cost of healthcare expenses, families in these nations are more inclined to have health insurance, which raises healthcare usage and improves the health of infants and young children. This ensures a sustained health outcome and insurance performance and contributes to growth. Benlagha and Hemrit [65] found that nonlife insurance activities are positively impacted by internet use but found no evidence of the demand for life insurance.

Another feature of structural transformation is urbanisation. Urbanisation increases insurable assets because new urban dwellers become more affluent and aspirational [8]. Zerriaa and Noubbigh [69] explain that a higher level of urbanisation is likely to increase life insurance demand in two respects. First, the concentration of consumers in a geographic area facilitates insurance distribution because urbanisation increases productivity, lowers the cost of goods, and increases the number of services available [8]. Second, a higher proportion of the urban population is associated with less reliance on informal insurance agreements, which may result in a higher demand for formal insurance products. Furthermore, urbanisation increases population density in urban areas, resulting in more interaction between people and a higher concentration of assets. As a result, urban residents perceive a higher risk of property damage, theft, and casualties, influencing individuals to purchase insurance products [70]. Hwang and Gao [71] discovered that urbanisation positively influences insurance penetration. They reveal the propensity of the urban population to save funds for retirement as one of the key reasons. However, Zerriaa and Noubbigh [69] failed to find any significant relationship between urbanisation and insurance. Canh et al. [72] also found a positive relationship and narrated that because urban residents have more assets, property, and liabilities that need to be covered, urbanisation may increase the intensity of insurance. Thus, the demand for insurance increases due to urbanisation, driving insurance penetration and bolstering economic performance. The arguments from the literature underscore the essential role of structural transformation in the insurance-growth nexus. However, empirical evidence to validate this relationship is hard to come by. Given the low insurance penetration rate and ongoing structural transformation in Africa, there is a need to explore how these factors interact to enhance economic growth, which is addressed in this study.

### 2.4 Gaps in the literature

The existing literature highlights that the relationship between insurance and economic growth has received considerable attention. Nonetheless, the inconsistent findings from the literature suggest that the relationship between growth and insurance is far from exhaustion. As a result, a comprehensive understanding of the conditions ensuring insurance market development is essential. As Horvey and Odei-Mensah [5] present, the performance of insurance on growth is contingent on several factors. Hence, to realise the full potential of insurance, one must consider these factors. However, prior studies mostly concentrate on the direct relationship between insurance and economic growth, with just a handful of scholars exploring the intervening role of ICT, terrorism, institutional quality and financial literacy in the relationship. As a result, the study reveals a gap in the literature on the potential impact of changes in the economic structure on this nexus. To this effect, this study addresses this gap by highlighting the importance of structural transformation in the insurance-growth nexus. As initially presented, the reallocation of resources from agriculture to other sectors, such as the industry and services sectors, known as structural transformation, has the potential to significantly modify the way insurance affects growth by modifying risk profiles, investment possibilities, and financial intermediation procedures. The novel approach not only broadens our knowledge of the relationship between insurance and growth but also offers insightful information about how changing economic structures can either strengthen or weaken the growth-promoting effects of insurance. As a result, policymakers will be able to view the issue from new angles and promote sustainable economic development. In addition, scholars provide insight into the long-run, short-run and bidirectional relationship between insurance and economic growth; notwithstanding, there has not been much discussion on the existence of non-linearities in the relationship, creating another vacuum in the literature. Therefore, this study explores the nonlinear effects of structural transformation and insurance on economic growth to understand if the impact varies at different threshold levels. This perspective holds that a well-developed insurance market and strong structural system are imperative for promoting economic growth. There is no better time to address this, given the renewed interest among African countries in structurally transforming their economies. To this end, this study fills an essential gap in the literature by examining the joint impact of insurance penetration and structural transformation on economic growth and determining the threshold level at which the interactions promote economic growth.

## 3 Method

### 3.1 Data

The study relies on annual secondary data spanning thirteen years from 2008 to 2020 to achieve the research objectives, using thirty-eight (38) economies^1^ in Africa for the analysis. The sample size and study period are exclusively based on data availability. Economic growth, the dependent variable, was measured by GDP growth (annual %), which captures GDP’s annual percentage growth rate. Data on insurance penetration were gathered from the Swiss Re Sigma database and measured as the total premiums as a percentage of GDP. The choice of this variable is consistent with the literature [7,17]. The moderator in this study is structural transformation, which was generated following [10,73,74]. This is further explained in subsection 3.2. We introduce other control variables on the grounds of econometric prudence. Foreign direct investment and trade were introduced to capture economic globalisation and to show how countries are open to the international market [51]. Inflation was used to capture the macroeconomic in/stability, and personal remittances refer to all transfers in cash or in kind received. Further details on the variable measurement and sources can be found in Table 1.

**Table 1:** Description of variables and sources.

Variable	Symbol	Definition	Source
***Dependent Variable***			
Gross Domestic Product	GDP	GDP Growth (annual %)	WDI
***Independent Variable***			
Insurance Penetration	IP	Premiums as a percentage of GDP	Swiss Re
***Moderator: Structural Transformation Variables (STI)***			
Agriculture	AGR	Agriculture, forestry, and fishing, value added (% of GDP)	WDI
Services	SER	Services, value added (% of GDP)	WDI
Manufacturing	MAN	Manufacturing, value added (% of GDP)	WDI
Industrial Sector	IND	Industry (including construction), value added (% of GDP)	WDI
Urbanisation	URB	Urban population (% of total population)	WDI
Infant mortality	Mortality	Mortality rate, infant (per 1000 live births)	WDI
Information and Communication Technology	ICT	Individuals using the Internet (% of population)	WDI
***Control Variables***			
Foreign Direct Investment	FDI	Foreign direct investment, net inflows (% of GDP)	WDI
Remittances	REMIT	Personal remittances, received (% of GDP)	WDI
Trade	TR	Trade (% of GDP)	WDI
Inflation	INF	Consumer price index (2010 = 100)	WDI

Note: WDI represents World Development Indicators.

### 3.2 Structural transformation index (STI)

The study constructs a multidimensional index to measure structural transformation using seven components as informed by the literature [10,73]. The index is based on four sectoral value additions (Industry, Agriculture, Manufacturing and Service sectors), two demographics (urbanisation and Infant mortality) and ICT developments [51]. An important feature of the structural transformation index must include the following: First, agriculture’s contribution to GDP must be decreasing. This is calculated by taking the inverse of its value additions [51]. This is characteristic of structural transformation because it represents the transition of African economies toward more diverse, productive and technologically advanced industries. Ibrahim et al. [10] state that heavy reliance on this sector is a deterrent to structural transformation; hence, there is a need for a shift from an agriculture-based economy to one with high-value-added industrial, manufacturing and service sectors.

Second, there must be an increase in productivity for the industrial, manufacturing and service sectors. This is computed by taking their respective growth rates [10,73]. An increase in these sectors is essential since structural transformation requires economies to move from being predominantly reliant on agriculture to becoming more centred on other sectoral activities. This will drive the overall economic output and allow for economic diversification. Again, these sectors facilitate the transformation of raw materials into finished products at various stages of production, generating higher value and revenue than primary sectors such as agriculture [74]. Third, there must be a rapid acceleration of urbanisation and improvement in ICT infrastructure. Urbanisation is incorporated to signify the quick changes in social structure as economies undergo structural change. Urbanisation also offers a wide range of benefits, such as employment and the development of skilled labour and ensures the development of infrastructure, which is essential for supporting industrial activities and attracting investments [51], all of which contribute to sectoral development. In addition, increased ICT infrastructure, like high-speed internet connectivity and modern communication networks, allows for the efficient flow of information, improves connectivity between individuals and enterprises, and facilitates digital transformation [5]. Fourth, there must be a lower mortality rate, which is critical to a country’s structural transformation program. The inclusion of this variable is to capture the inclusiveness of the structural transformation process [73]. It indicates a country’s general health and well-being, particularly regarding maternal and child health.

This study follows the methodological approach of Ibrahim et al. [10] and Armah and Baek [73] to construct the structural transformation index. Principal component analysis was adopted to create the index due to its ability to group different variables into a single composite index.^2^ The index was then normalised to range between 0 and 1. This technique helps to assess the country with high or low structural transformations. The normalisation was done using the Min-Max normalisation procedure as specified in equation (1).

(Eq. 1) $${STI}_{it}=\left(X_{it}-X_{\min}\right)/\left(X_{\max}-X_{\min}\right)$$

Such that $X_{\max}\;and\;X_{\min}$ represents the maximum and minimum values of the initial index for the respective countries across the years; $X_{it}$ represents the value of the initial index in country *i* at time *t*. As a result, the normalised indicators range between 0 and 1. Hence, the higher the STI, the higher the structural transformation and vice versa.

### 3.3 Empirical strategy

#### 3.3.1 Linear and interaction effects

This study begins the analysis by examining the direct and synergistic relationship between insurance, structural transformation and economic growth by employing the dynamic generalised method of moments (GMM) estimator proposed by Arellano and Bond [75]. One of the primary motivations for selecting this technique is based on the caveat that the number of cross-sections (i.e., countries) must be greater than the time periods, which is the case in this study, where the number of countries (thus N = 38) is greater than time (thus T = 13). More so, this technique is appropriate because it can control endogeneity problems, omitted variables, and measurement errors by exploiting the data’s time series variations and controlling for unobserved country-specific effects [76]. It is important to note that including the lagged term of the dependent variable in the regression model may result in endogeneity problems because the lagged dependent variable is dependent on the lagged error term, which is a function of the country-specific fixed effects. Further, we also suspect endogeneity bias due to the reverse causality between insurance penetration and economic growth [20,21,77,78]. Against these backdrops, we set a model where the dependent variable depends on its lag and a vector of observations of the independent variables, thereby validating the use of the dynamic GMM estimator. Further, a two-step GMM is employed instead of the one-step GMM because it controls heteroscedasticity and autocorrelation while capturing omitted variable problems and measurement errors. As a result, the regression model is specified as follows:

(Eq. 2) $${GDP}_{it}={\beta_{1}GDP}_{it-1}+{\beta_{2}INS}_{it}+{\beta_{3}FDI}_{it}+{\beta_{4}REMIT}_{it}+{\beta_{5}TR}_{it}+{\beta_{6}CPI}_{it}+\mu_{i}+\partial_{t}+\varepsilon_{it}$$

(Eq. 3) $${GDP}_{it}={\beta_{1}GDP}_{it-1}+{\beta_{2}ST}_{it}+{\beta_{3}FDI}_{it}+{\beta_{4}REMIT}_{it}+{\beta_{5}TR}_{it}+{\beta_{6}CPI}_{it}+\mu_{i}+\partial_{t}+\varepsilon_{it}$$

(Eq. 4) $${GDP}_{it}={\beta_{1}GDP}_{it-1}+{\beta_{2}INS}_{it}{+\beta_{3}ST}_{it}+{\beta_{4}\left(INS*ST\right)}_{it}+{\beta_{5}FDI}_{it}+{\beta_{6}REMIT}_{it}+{\beta_{7}TR}_{it}+{\beta_{8}CPI}_{it}+\mu_{i}+\partial_{t}+\varepsilon_{it}$$

Where GDP represents gross domestic product; ${GDP}_{it-1}$ represents the one-period lag of the dependent variable; INS represents insurance penetration; ST represents the structural transformation index and its individual components; FDI represents foreign direct investment; REMIT represents remittances, TR represents trade; CPI represents consumer price index; β represents the parameters to be estimated; $\mu_{i}$ represents the unobserved country-specific fixed effects; $\partial_{t}$ represent the time fixed-effect and $\varepsilon_{it}$ is the idiosyncratic error term.

With respect to the interaction between insurance penetration and structural transformation, the study follows the conditions specified by Brambor et al. [79] to test for the joint significance of the constitutive and interactive terms to arrive at the net effect of insurance penetration and structural transformation on economic growth. As a result, the net effect was derived by taking the first derivative of the constitutive and interactive terms with respect to insurance penetration in equation (4). Given this, our model is specified as follows:

(Eq. 5) $$Net\;Effects:\frac{\partial GDP}{\partial INS}=\beta_{2}+\beta_{4}{\text{ST}}_{\text{it}}=0$$

Hence, the level of insurance penetration interacted by structural transformation is examined by the summation of the coefficients ($\beta_{2}+\beta_{4}$) and the test of the significance of the joint effects.

The study highlights that the validity and reliability of the GMM results are based on the following assumptions. First, there must be a first-order serial correlation. Second, there must be no second-order serial correlation. Third, the Hansen test of overidentifying conditions must be statistically insignificant to ensure the validity of the instruments. Thus, the null hypothesis must not be rejected. Fourth, the inclusion of the lagged dependent variable must be statistically significant to affirm the dynamic nature of the model [80]. These assumptions are checked when interpreting the results to ensure their accuracy and reliability.

#### 3.3.2 Nonlinear relationship

The study further explored the threshold level at which the interactive and constitutive terms estimated in equation (4) affect economic growth using the dynamic panel threshold estimation technique [81,82]. The dynamic panel threshold approach is chosen for this study because it is built on GMM principles, which deal with endogeneity and simultaneity issues [83]. It also extends the threshold model by Hansen [84], which is particularly useful for static models and Kremer et al. [85]. More so, these models do not provide comprehensive information on the direction of the coefficient estimates of the variables at different thresholds, a limitation addressed by the dynamic panel threshold model, offering more comprehensive information by indicating the effect of the coefficient estimates at different levels [1,82]. It also helps to capture the nonlinear effects of the interaction term on economic growth [80]. The threshold effects are determined by dividing the determinants into upper and lower regimes. The use of this technique is to determine the level of insurance penetration and structural transformation required to stimulate economic growth. This technique assumes a specific threshold level at which the relationship between insurance penetration, structural transformation, and economic growth changes. This assumption implies that the relationship is nonlinear and that the effects of insurance penetration differ based on whether it is above or below the threshold. The technique assumes that the threshold variable, insurance penetration in this case and explanatory variable, is endogenous and not influenced by the error term in the regression equation. Hence, the model is specified as:

(6) $$y_{it}=x_{it}^{\prime }\beta +\left(1,x_{it}^{\prime }\right)\delta 1\left\{q_{it}>\gamma \right\}+\mu_{i}+\varepsilon_{it}\;i=1,\ldots ,n;\;t=1,\ldots ,T$$

Where: $y_{it}$ is the dependent variable, which is economic growth $q_{it}$ represents the threshold variable, splitting the sample into two regimes, which is an indicator of insurance penetration, the main independent variable and $x_{it}$ includes the lagged dependent variable and control variables, γ is the threshold parameter. T is assumed to be fixed, whereas n is assumed to grow indefinitely. 1{.} is the indicator function specifying the regime. The fixed effect $\mu_{i}$ is removed using the first difference process, and then the unknown parameters are estimated using GMM. Thus θ = $\left(\beta^{`},\delta^{`},\gamma \right)$.

## 4 Findings and discussions

### 4.1 Summary statistics

We present our analysis by starting with the summary statistics to provide a brief overview of the variables included in the regression model. The outline of the dataset is presented in Table 2. The data shows an average GDP growth (annual %) value of 3.799. The standard deviation value is 4.577, showing a relatively small variation. Insurance penetration recorded an average value of 1.657 and did not show much variability across the countries. This average score indicates that there has been a low contribution of insurance to GDP growth over the years in Africa. We also observe an average structural transformation index (STI) score of 0.459 over the study period, implying less structural transformation in Africa. The results of the correlation analysis imply no presence of multicollinearity.

**Table 2:** Descriptive statistics and correlation matrix, 2008–2020.

	GDP(1)	Insurance(2)	STI(3)	FDI(4)	REMIT(5)	Trade(6)	Inflation(7)
**Obs**	494	494	494	494	494	494	494
**Mean**	3.799	1.657	0.459	4.574	3.201	74.195	134.555
**Std. Dev.**	4.577	2.31	0.070	6.507	4.729	34.838	125.487
**Min**	−20.599	0.004	0.000	−11.199	0.000	20.722	75.731
**Max**	20.716	14.26	0.998	57.877	32.593	225.023	2725.31
**Q1**	1.996	0.510	0.428	1.385	0.362	50.029	103.482
**Q2**	4.362	0.837	0.452	2.887	1.563	64.950	113.482
**Q3**	6.256	1.756	0.483	5.248	3.665	92.139	138.851
**Correlations**							
**(1) GDP**	1						
**(2) Insurance**	−0.155∗∗∗	1					
**(3) STI**	0.241∗∗∗	−0.059	1				
**(4) FDI**	−0.021	−0.057	0.071	1			
**(5) REMIT**	−0.017	0.036	0.039	−0.081∗	1		
**(6) Trade**	−0.119∗∗∗	0.160∗∗∗	−0.005	0.421∗∗∗	0.154∗∗∗	1	
**(7) Inflation**	−0.144∗∗∗	0.028	0.056	−0.071	0.049	−0.068	1

**Note:** (∗), (∗∗) and (∗∗∗) highlights significance at 10 %, 5 % and 1 %, respectively; Q1, Q2, and Q3 represents the 25^th^, 50^th^ and 75^th^ percentiles respectively. **Source:** Results were generated from Stata by authors.

**Fig. 1:**
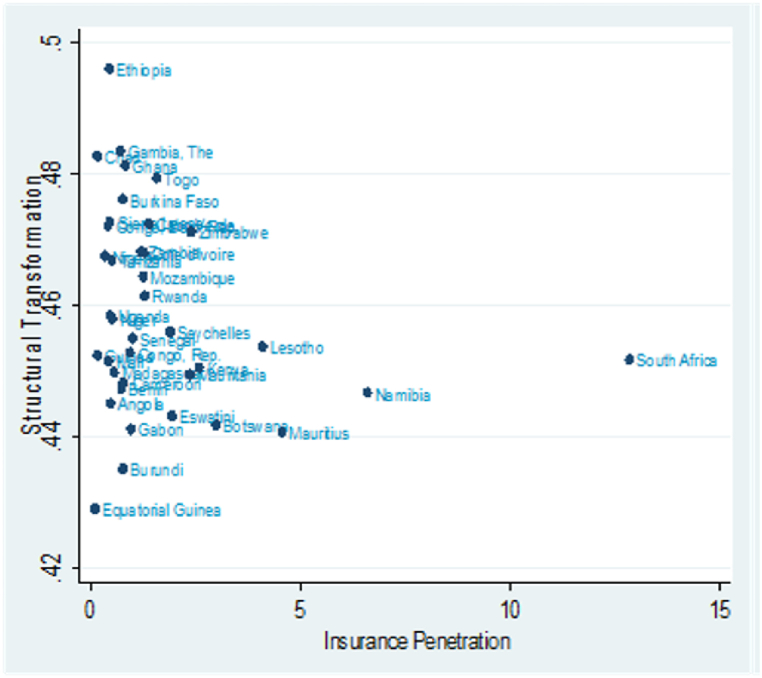
Scatter plots of Insurance Penetration and Structural Transformation.

**Fig. 2:**
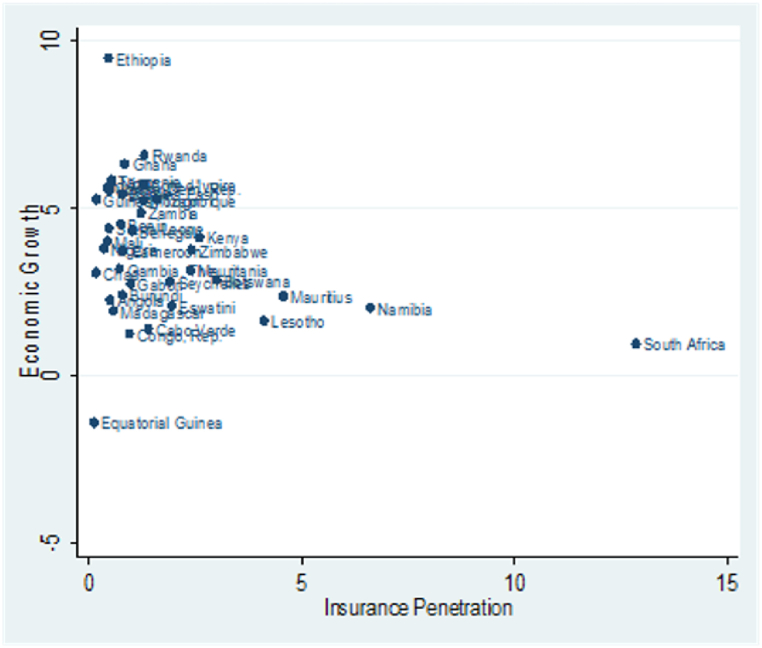
Scatter plots of Insurance Penetration and Economic Growth.

Again, we present the scatter plots in Fig. 1, Fig. 2 to provide a cursory view of the association between insurance penetration, structural transformation, and economic growth averaged 2008–2020 with one observation for each country. In Fig. 1, insurance penetration and structural transformation distribution appear homogeneous. Similarly, the distribution of insurance penetration and economic growth are also homogeneous, as illustrated in Fig. 2. However, some outliers are noticeable. For instance, South Africa records the highest insurance penetration, while Equatorial Guinea has the lowest insurance penetration with a relatively low structural transformation index and GDP growth. On the contrary, Ethiopia has the highest structural transformation index and GDP growth.

### 4.2 Regressions results

#### 4.2.1 The direct effects of insurance and structural transformation on economic growth

The empirical results obtained from using the two-step system GMM are reported in this section. Table 3 presents the findings of the direct impact of insurance and structural transformation on economic growth. The conditions for using GMM are met in both tables. There is no presence of second-order serial correlation, and the insignificance of the Hansen test for over-identifying restrictions confirms the joint validity of the internal instruments of the model. The lag of GDP growth is statistically significant at 1 % across all model specifications, indicating the persistence of GDP growth in Africa. This also suggests a divergence in economic growth in Africa. The implication is that countries with high initial GDP levels will continue to experience higher growth in their current year compared to those with lower growth. This is consistent with prior literature suggesting that past GDP growth strongly predicts its current level [21,50]. This indicates that time matters in explaining economic growth in Africa; thus, a country’s level of economic growth in the current year reinforces its level of growth in the subsequent year.

**Table 3:** Direct effects of insurance and structural transformation on economic growth (Dep Var: GDP growth).

	(1)	(2)	(3)	(4)	(5)	(6)	(7)	(8)	(9)
Lag of GDP	0.267∗∗∗	0.245∗∗∗	0.271∗∗∗	0.275∗∗∗	0.272∗∗∗	0.266∗∗∗	0.293∗∗∗	0.261∗∗∗	0.245∗∗∗
	(0.091)	(0.085)	(0.087)	(0.086)	(0.096)	(0.081)	(0.063)	(0.072)	(0.087)
FDI	−0.424	−0.485	−0.552	−0.423	−0.443	−0.343	−0.329	−0.280	−0.485
	(0.397)	(0.391)	(0.443)	(0.398)	(0.459)	(0.315)	(0.212)	(0.279)	(0.391)
Remittances	−0.976	−1.043∗	−0.992∗	−0.965	−0.987	−0.818	−0.498	−0.600	−1.043∗
	(0.699)	(0.586)	(0.520)	(0.783)	(0.765)	(0.623)	(0.344)	(0.469)	(0.586)
Trade	0.081	0.084	0.143∗	0.090	0.078	0.078	0.036	0.034	0.084
	(0.068)	(0.066)	(0.073)	(0.068)	(0.077)	(0.057)	(0.045)	(0.054)	(0.066)
Inflation	−0.018∗∗	−0.019∗∗	−0.014∗∗	−0.017∗∗	−0.019∗∗	−0.013∗	−0.010∗∗	−0.011∗∗	−0.019∗∗
	(0.008)	(0.008)	(0.006)	(0.008)	(0.009)	(0.007)	(0.004)	(0.005)	(0.008)
Insurance	0.026								
	(0.042)								
Industry		7.353∗∗							
		(3.100)							
Agriculture			−3.108∗∗						
			(1.152)						
Manufacturing				4.111∗					
				(2.430)					
Service					5.818∗∗				
					(2.950)				
Urbanisation						0.064			
						(0.139)			
ICT							0.096∗∗∗		
							(0.031)		
Mortality								0.166∗∗∗	
								(0.060)	
Structural transformation index									2.434∗∗∗
									(0.419)
Year and Country Dummies	Yes	Yes	Yes	Yes	Yes	Yes	Yes	Yes	Yes
Observations	455	455	455	455	455	455	455	455	455
Number of groups	38	38	38	38	38	38	38	38	38
Number of instruments	11	11	11	11	11	11	11	11	11
Prob > F	0.000	0.000	0.000	0.000	0.000	0.000	0.000	0.000	0.000
AR(1)	0.000	0.000	0.001	0.000	0.001	0.001	0.000	0.000	0.000
AR(2)	0.382	0.406	0.214	0.453	0.415	0.366	0.330	0.411	0.406
Hansen	0.165	0.141	0.114	0.122	0.202	0.096	0.265	0.310	0.141

Note: Standard errors in parentheses; Significant levels are expressed as ∗∗∗p < 0.01, ∗∗p < 0.05, ∗p < 0.1, respectively; Where GDP represents economic growth; FDI represents foreign direct investment; ICT represents information and communication technology.

We begin our analysis by first exploring the direct effect of insurance on economic growth in Africa, as shown in Model (1). The result documents a positive relationship between insurance and economic growth, suggesting that the development of the insurance market is essential to economic growth. Several studies from the literature support this finding [6,16,63]. For instance, Ali [39] argues that insurance drives the growth of economies by creating a capital base and financial security for businesses. Balcilar et al. [17] support this assertion by narrating that insurance provides loss indemnity, saving mobilisation, and investment possibilities, ensuring business stability and promoting economic growth. Despite the positive relationship, the result is statistically insignificant. This is explained by Outreville [22], who suggests that though insurance is an important factor in the growth of economies, its impact varies depending on the insurance market’s maturity, which might explain the insignificant result. Further, the results align with the reality of the insurance market in Africa, which has a penetration rate of about 2 %, remaining the lowest globally, suggesting that the insurance market in Africa is not mature enough to realise its growth potential fully. Horvey and Odei-Mensah [5] support this assertion by explaining that the insurance market in Africa is still growing and is affected by several regional issues, such as poverty, high unemployment, illiteracy levels and income inequality. Hence, when a high percentage of the population has limited disposable income, it is difficult for individuals to obtain insurance coverage, which might explain the low penetration and insignificant result. Asongu and Odhiambo [21] add that insurance policies have not been effectively adapted to African needs and realities because these insurance operations are based on complex and protracted contracts and are distributed through expensive networks of brokers and agents that are largely restricted to urban elites and areas. The lack of context-specific policies restricts the acceptability and coverage of the insurance market, which in turn affects the industry’s performance. As a result, there is a need for a strong and well-developed insurance industry that can significantly boost economic growth [36]. This requires the consideration of context-specific demands and other environmental conditions and structures important to driving the insurance market [15]. Thus, when the insurance market is developed considering other contextual factors, its propelling effect on economic growth will be realised.

Further, the study examines the direct impact of the structural transformation index and its individual variables to understand how each reacts to economic growth before launching into their moderating influence. The empirical evidence in Model (9) illustrates a positive relationship between the structural transformation index and economic growth. The relationship is statistically significant, implying that countries that undergo structural transformation achieve higher growth, affirming the argument that structural transformation is pivotal in driving economic growth [9,50]. This is not surprising, given that structural change enhances productivity across the economy by shifting from low-productivity sectors to those with higher and more diverse productivity [12]. Thus, overall efficiency increases as resources are transferred from low-productivity to high-productivity sectors [9]. Other benefits of structural transformation include economies of scale, technical innovation, and specialisation, which contribute to an increase in productivity levels, improvement in income distribution levels and promotion of a well-diversified and resilient economy [74].

Considering the individual structural transformation factors, the study reveals that the industrial and service sectors positively influence economic growth. The positive relationship is plausible given that these sectors drive innovation, value addition, job creation and higher productivity [86,87]. An increase in productivity in these sectors leads to a more efficient use of resources, which speeds up economic growth [62]. This affirms their importance to the economic productivity of countries. Similarly, the manufacturing sector positively impacts economic growth, which can be attributed to the fact that the manufacturing sector mostly takes the lead in technical innovation and productivity gains [74]. More so, research and development (R&D) and industrial technology investments in this sector promote efficiency benefits and encourage innovation, which can positively affect the economy. On the contrary, agriculture presents a negative impact, showing that it weakens economic growth. These findings align with the Kuznet hypothesis that as economies change over time from being heavily dependent on agriculture to being more industry- and service-oriented, the impact of the agriculture sector on growth reduces while the impact of other sectors improves [88]. Hence, sustained economic growth is seen in the change that is being accompanied by an increase in per capita income and better living conditions [88]. Given the above, governments should create industrial policies that promote the expansion of manufacturing and other sectors by providing focused incentives, funding infrastructural projects, and establishing business-friendly regulatory frameworks.

Further, the results show that urbanisation positively influences economic growth. Hong et al. [89] support this finding by explaining that urban areas have developed infrastructure, skilled labour, and easy access to cutting-edge technology. This encourages specialisation, division of labour, and competitive advantage, as well as increasing productivity and fostering growth. Additionally, urban areas draw a wide range of talent and offer more opportunities to align qualifications with job requirements [8]. This reduces underemployment and unemployment rates, increases labour market efficiency and raises total productivity and economic output [87]. More so, highly concentrated economies, in which the closeness and density of economic activity promote efficiency and creativity, are frequently enabled by urban areas. Cities with a concentration of businesses, industries, and skilled labour foster information diffusion, teamwork, and specialisation, all of which boost competitiveness and economic productivity [74]. The finding further presents that ICT and mortality exhibit a significant positive impact on economic growth. ICT boosts productivity in several businesses by facilitating more effective communication, lowering transaction costs, and streamlining corporate operations [5]. It encourages improved coordination, automates tedious tasks, and real-time information sharing among supply chain stakeholders. Also, lower mortality rates are associated with better life expectancy and overall health outcomes. Healthy people are more likely to enter the labour market, which leads to a bigger and more productive workforce. This increasing labour force participation encourages human capital development because it makes economic activity and innovation more accessible to more individuals [73].

#### 4.2.2 The synergistic effects of insurance and structural transformation on economic growth

Taking the discussion further, this study explores the intervening role of structural transformation on the insurance-growth nexus. The results are reported in Table 4. Models (10)–(16) present the findings of each structural transformation variable and their interactions with insurance penetration. Model (17) reports the results of the interaction between the structural transformation index and insurance penetration. The empirical result reveals a significant positive interaction between the overall structural transformation index and insurance penetration in affecting economic growth. This is in line with the arguments by Lee and Chang [54], who point out that structural change increases the benefits of insurance by presenting various opportunities to utilise insurance for investment and risk management. Insurance helps companies handle specific risks, which in turn promotes the growth of more capital-intensive businesses and the economy. This suggests that structural change improves the financial benefits of insurance services and creates an atmosphere more conducive to insurance market development [41]. This indicates the critical role of structural transformation in enhancing the impact of insurance on growth. The study, therefore, argues that the pattern of structural change is vital to the insurance market. This aligns with Geda et al.’s [12] argument that structural transformation enhances industrial and financial development. The net effects also present positive results, indicating that strong structural transformation heightens the impact of insurance on economic growth. This finding makes economic sense for several reasons. For instance, the availability and efficient structural set-up improve the standard of living and enhance the insurance market [14]. Hence, the region’s high structural transformation level will strengthen insurance performance by encouraging adaptability to market conditions, improving innovation and customer experience, raising efficiencies, improving access and distribution channels, and enhancing trust and credibility, eventually positively impacting economic growth. Given the findings of this study, it can be argued that improved structural transformation will have an equalising effect on insurance penetration, accelerating economic growth. As a result, Kedir et al. [57] suggest that significant funding and government contributions are needed to achieve the desired structural transformation outcomes.

**Table 4:** The interaction effect of insurance and structural transformation on Economic Growth (Dep Var: GDP growth).

Variables	(10)	(11)	(12)	(13)	(14)	(15)	(16)	(17)
Lag of GDP	0.268∗∗∗	0.281∗∗	0.256∗∗∗	0.282∗∗∗	2.389∗∗	3.125∗∗	0.233∗∗∗	0.247∗∗∗
	(0.080)	(0.109)	(0.082)	(0.091)	(0.958)	(1.663)	(0.077)	(0.080)
Insurance	0.014	0.504∗∗	0.093	0.048	2.871	1.561	2.298	1.102∗∗
	(0.039)	(0.184)	(0.099)	(0.061)	(5.275)	(1.067)	(5.579)	(1.398)
FDI	−0.475	−0.785	−0.459	−0.488	1.143	0.296	−0.401	−0.415
	(0.357)	(0.521)	(0.388)	(0.497)	(0.920)	(0.255)	(0.373)	(0.346)
Remittances	−0.994∗	−1.087	−0.937	−0.990	1.294	0.329	−0.739∗	−0.874∗
	(0.535)	(0.714)	(0.568)	(0.703)	(1.078)	(0.481)	(0.468)	(0.415)
Trade	0.088	0.140	0.089	0.067	−0.068	−0.001	0.060	0.069
	(0.062)	(0.092)	(0.067)	(0.080)	(0.147)	(0.060)	(0.067)	(0.058)
Inflation	−0.017∗∗	−0.019∗∗	−0.018∗∗	−0.020∗∗	0.024	0.016∗	−0.012∗	−0.019∗∗
	(0.007)	(0.009)	(0.008)	(0.010)	(0.019)	(0.008)	(0.007)	(0.008)
Industry	−0.282							
	(1.030)							
Insurance∗Industry	4.399∗∗							
	(1.967)							
Agriculture		−4.254∗∗						
		(1.945)						
Insurance∗Agriculture		5.739∗∗∗						
		(2.119)						
Manufacturing			−5.064					
			(2.998)					
Insurance∗Manufacturing			0.743					
			(0.518)					
Service				4.859∗∗				
				(1.145)				
Insurance∗Service				1.076				
				(1.840)				
Urbanisation					−0.584			
					(0.356)			
Insurance∗Urbanisation					0.048			
					(0.071)			
ICT						−0.097		
						(0.093)		
Insurance∗ICT						0.015∗∗		
						(0.006)		
Mortality							0.319∗∗	
							(0.138)	
Insurance∗Mortality							−0.113	
							(0.086)	
STI								−0.438
								(0.434)
Insurance∗STI								2.841∗∗
								(1.059)
Net Effects	0.041∗	1.223∗∗	0.102	0.057	1.930∗∗	1.828∗	2.657∗	2.406∗∗
Year and Country Dummies	Yes	Yes	Yes	Yes	Yes	Yes	Yes	Yes
Observations	455	455	455	455	455	455	455	455
Number of groups	38	38	38	38	38	38	38	38
Number of Instruments	13	13	13	13	13	13	13	13
Prob > F	0.000	0.000	0.000	0.000	0.000	0.000	0.000	0.000
AR(1)	0.000	0.005	0.000	0.001	0.004	0.003	0.000	0.000
AR(2)	0.428	0.216	0.728	0.475	0.820	0.575	0.643	0.428
Hansen p-value	0.146	0.389	0.161	0.239	0.187	0.076	0.414	0.146

**Note:** Standard errors are in parentheses; Significant levels are expressed as ∗∗∗p < 0.01, ∗∗p < 0.05 and ∗ p < 0.1, respectively. Where GDP represents economic growth; FDI represents foreign direct investment; ICT represents information and communication technology; STI represents the overall structural transformation index. **Source:** Results were generated from Stata by authors.

Considering the individual structural transformation variables, the industrial sector presents a significant positive interaction with insurance penetration. The positive net effect further affirms this. This suggests that growth in the industrial sector can amplify the impact of insurance on economic growth. This is explained by Prokopjeva et al. [14], who present that the industrial sectors confront various risks in addition to the regulatory frameworks that impose obligatory insurance needs, which can greatly increase insurance penetration rates, thereby affecting economic growth. As a result, they are more likely to rely on insurance to cater for most of their operations, including casualty and fire insurance and risk management services [62]. The manufacturing sector also reveals an insignificant positive interaction and net effect. The positive relationship aligns with Banker et al.’s [60] position that the manufacturing industry is capital-intensive with an array of operational risks; hence, there is a significant need for various insurance services. Given this, the manufacturing companies require broad insurance coverage as they expand to lower the risks of equipment breakdowns, supply chain disruptions and production delays. Further, the insignificant results explain that this sector does not display any considerable contentious impact on insurance penetration. One possible reason for this may be due to the low manufacturing output in the region [11]. As it stands, Africa’s contribution to the global manufacturing output is around 2 %. This difference is reflected in the trade deficit, where manufactured products account for 62 % of all imports, which is likely to explain why this variable does not significantly influence the performance of insurance on growth.

Similarly, the service sector positively moderates the insurance-growth nexus. According to Sare et al. [63], the service sector usually requires specialised insurance coverage, including cyber insurance, professional liability coverage and insurance against malpractices to reduce operational risks. This drives the need for more insurance products, ultimately enhancing insurance performance and its impact on economic growth. Despite the important role of the service sector in insurance market development, the study finds that its effect is not substantial due to the insignificant result. This is explained by Ibrahim et al. [10], who posits that there is a high-income differential in the service sector in Africa, which comprises many low-income earners where most residents work informally, primarily by selling daily necessities on the street and with few high-income earners with some employees working in industries including real estate, banking, transportation, and information technology. To ensure their significant influence on insurance, the study suggests that African countries must encourage the industrialisation and development of the service and manufacturing sectors [10]. Also, these sectors need to engage the insurance industry for knowledge-sharing on issues such as risk management, business interruption, and tax subsidies. This will safeguard and promote the insurance industry’s growth, thereby significantly improving insurance penetration. Governments can also play a crucial role by ensuring that these sectors purchase insurance products such as liability insurance, worker’s compensation, and fire insurance to support their business operations [62].

Further, the agriculture sector positively and significantly moderates the insurance-growth nexus. As much as structural transformation requires a transition from the agriculture sector to industrialisation, this sector remains dominant in most African countries and is mostly susceptible to risk, including pests and erratic weather, which likely affects their revenue and productivity. This raises the need for insurance to ensure productivity and to invest in modern technology and efficient farming practices [59]. The significant positive result is not surprising given that agriculture remains a pillar of most African countries. As reported by Statista, the agriculture sector accounted for over 17 % of Africa’s GDP in 2021, with Sierra Leone being the highest contributor (60 %). This is followed by Chad (54 %) and Ethiopia (38 %). Thus, there is a need to foster collaborations between agriculture and insurance to develop tailor-made insurance products that address these sectors' needs. More so, the result displays that ICT development significantly induces insurance performance on growth. This is because technological advances help insurers foster innovation and investment for economic growth [5]. It also provides direct distributional systems and easy customer communication [64]. Pradhan et al. [3] narrate that increased accessibility to smartphones, the internet, Internet of Things, sensor technologies and satellite communications provides the insurance industry with more data and makes them more informative in their decisions and policy offerings. This helps insurers to keep up with the pace of structural change. As a result, insurers better understand their customers and use customer data to design more relevant products and better risk pricing, providing better underwriting services. Again, ICT provides opportunities for insurers to remain connected with policyholders by receiving and responding to their demands through digital devices. This also provides avenues for insurers to operate on a global scale since clients do not need to be physically present to purchase insurance products [65]. These provide a foundation for insurance market development, which in turn affects economic growth.

Additionally, the study finds evidence that urbanisation strengthens the performance of the insurance industry. The result could be attributed to the fact that the concentration of consumers in a geographic area facilitates insurance distribution because urbanisation increases productivity, lowers the cost of goods, and increases the number of services available [8]. Thus, urbanisation facilitates insurance distribution, which may result in a higher demand for formal insurance products [69]. Also, urban settlers perceive higher risks of property damage, theft, and casualties. Therefore, they look for ways, such as insurance, to mitigate urban risks [70]. This is supported by Canh et al. [72], who narrate that because urban residents have more assets, property, and liabilities that need to be covered, this results in an increase in the intensity of insurance. Thus, the demand for insurance increases due to urbanisation, increasing insurance penetration and bolstering economic performance. Infant mortality revealed a negative moderating influence but produced a positive net effect. The results show that insurance improves economic growth through these intervening factors. That is, these factors heighten the impact of insurance penetration on economic growth. According to the literature, there is an association between low infant mortality and insurance. For instance, the World Bank indicated that the infant mortality rate in Africa, as of 2021, was close to 51 % [90]. This has prompted governments in Africa to initiate healthcare and social welfare programs, such as insurance schemes, in response to the high infant death rates. As a result, countries such as Ghana, Kenya, Mali, Nigeria, Rwanda, South Africa, Tanzania and Zimbabwe have implemented insurance schemes to improve health outcomes and infant mortality. By implementing these measures, insurance penetration is enhanced, which is affirmed by the positive results, promoting economic growth.

With the controls, the study reveals that foreign direct investment, remittances, and inflation hurt economic growth. In Africa, issues such as lack of regulatory frameworks, poor governance, corruption, and inadequate institutional capacity make it difficult for African nations to fully reap the rewards of FDI; hence, the reason behind the negative impact. For inflation, Alagidede et al. [50] contend that higher inflation rates reallocate scarce resources to unproductive activities, lowering economic growth. Again, trade shows a positive effect, suggesting that trade openness promotes economic growth because it increases labour productivity [91]. This is very true in Africa, where production is more import-driven. Trade openness helps firms to have access to importing productive inputs to increase productivity and enhance sectoral value and economic growth [51]. Hence, we suggest that further openness to international trade will help spur economic growth in Africa.

#### 4.2.3 Threshold analysis of structural transformation, insurance and economic growth

Another contribution of this study is the examination of nonlinearities in the relationship. As a result, the interaction between the overall structural transformation index and insurance penetration is explored to determine its impact at different thresholds, as reported in Table 5. The linearity test was significant at 1 %. Therefore, we reject the null hypothesis of no threshold effect, confirming nonlinearity in the model. Hence, our sample can be split into two regimes. The empirical evidence also suggests that the minimum threshold level of insurance penetration estimated to achieve economic growth is 2.12 %. Below the threshold level, insurance penetration is significantly negative. We also find a significant positive relationship in the upper regime, implying a U-shaped relationship. This suggests that high insurance penetration is vital to improving economic growth in Africa and vice versa. This argument is supported by Asongu and Odhiambo [21]. This is because low insurance penetration suggests a lack of financial security, which could impede the expansion and stability of the economy. Thus, economic operations may be less safe and more vulnerable to risks if there are insufficient insurance products or individuals do not have insurance coverage, which would have a detrimental effect on growth [4]. On the other hand, higher insurance penetration levels reduce financial uncertainty, promote investment and support trade, commerce and entrepreneurial activity [3]. This supports Apergis and Poufinas [37], who state that a strong insurance market is imperative for the growth and development of economies. Zulfiqar et al. [38] add that insurance provides financial security to individuals and businesses, which promotes growth and investment, leading to overall economic performance. In this regard, the study posits that economic growth is enhanced when insurance penetration exceeds a certain threshold level. Hence, African countries should consider measures to develop the insurance market in order to realise its growth potential.

**Table 5:** Threshold Model with Interaction Variable (Dep Var: GDP growth).

VARIABLES	Lower Regime		Upper Regime
Lag of GDP	0.355∗		0.115∗∗∗
	(0.234)		(0.006)
Insurance	−1.013∗		2.921∗∗
	(0.654)		(1.115)
Insurance∗STI	−2.717		3.197∗∗
	(1.984)		(1.540)
STI	0.177		1.084∗∗∗
	(0.190)		(0.106)
FDI	−0.155∗∗		0.362∗∗∗
	(0.070)		(0.026)
Remittances	−1.265		1.673∗∗
	(1.014)		(0.749)
Trade	−0.065		0.197∗∗∗
	(0.105)		(0.031)
Inflation	−0.003∗		0.001
	(0.001)		(0.000)
***Post-estimations***			
Threshold Value		2.121∗∗	
		(1.042)	
Linearity Test (P-value)		0.000	
Countries		38	

**Note:** Standard errors are in parentheses; Significant levels are expressed as ∗∗∗p < 0.01, ∗∗p < 0.05 and ∗ p < 0.1, respectively. Where GDP represents economic growth; FDI represents foreign direct investment; and STI represents the overall structural transformation index. **Source:** Results were generated from Stata by authors.

Further, the results for structural transformation reveal a positive effect on economic growth for both regimes, demonstrating a significant positive impact on economic growth in the upper regime, implying that high structural transformation improves economic growth [26]. The insignificant result in the lower regime suggests that low structural transformation is not strong enough to present any considerable impact on economic growth. This is because, in the initial phases of structural change, an economy could expand more slowly and experience some disruptions in the transformation process, including job losses, inefficiencies and the need for new infrastructure and skills [73,88]. However, as the structural transformation process becomes more advanced and developed, it provides resilience and can sustain economic growth [50]. Additionally, the analysis presents a significant negative interaction effect between insurance and structural transformation in the lower regime. This illustrates that low structural transformation hinders the insurance industry’s development, eroding economic growth (Africa Re, 2020). This is affirmed by Horvey and Odei-Mensah [5], who highlight that structural inefficiencies can dampen the development of the insurance market and its growth-lubricating effect. They further claim that in countries with advanced structural transformation, insurance services may be able to help the industrial and service sectors more successfully.

However, in the upper regime, it can be seen that the interaction between insurance penetration and structural transformation presents a significant positive impact on economic growth, implying that high structural transformation levels advance the effect of insurance on growth [8,55]. This suggests that the influence of insurance penetration on economic growth is contingent on the relative development of structural transformation. In other words, increasing structural transformation stimulates insurance penetration’s impact on economic growth. This is plausible because countries undergoing structural transformation require insurance services to safeguard their investment, innovation and stability accompanying industrialisation. Hence, the increasing demand for insurance provokes the industry’s growth, which in turn drives the growth of economies [3]. More so, higher structural transformation leads to technological advancements in data models and analytics, improving risk detection and quantification [66]. This gives insurers more accurate information for risk analysis and insurance, leading to higher penetration. Improved structural transformations, such as increased manufacturing, service, and industrial sectors, often involve higher risks and business interruptions, which require insurance to protect against these losses [14]. These requirements enhance insurance penetration in these sectors, thereby fostering economic growth. Again, structural transformation elevates the desire for risk management and financial security, increasing the demand for insurance policies. This ensures sustainability, investment, improved market efficiency and economic growth.

The economic implication of this study is that for insurance to significantly influence economic growth, African countries must improve their level of structural development. This argument is affirmed by PwC [8], stating that structural transformation is fundamental to the development of the insurance markets. Investment in structural transformation policies is crucial to boost economic growth and the insurance market in Africa, particularly in ensuring the movement of labour from low-productivity to high-productivity sectors. Again, countries implementing policy-based agriculture insurance might heighten farmers’ interest in participating in insurance by providing premium subsidies [59].

### 4.3 Robustness check

The study’s findings are cross-validated by using another proxy for economic growth, GDP per capita. This is often used in empirical literature to explain the level of economic development or a country’s economic output per person [5]. The results are shown in the appendix (see Tables 6–8). Like Table 4, insurance penetration and structural transformation interaction confirm a positive result. The individual factors also show positive net effects on economic growth. The dynamic threshold results in Table 8 also display similar coefficients below and above the threshold value. Thus, in both cases, our findings suggest that the impact of insurance on economic growth is greater at higher levels of structural transformation, affirming the robustness of the results. As a result, the findings are reliable and useful for inferences.

## 5 Conclusion and policy implications

### 5.1 Summary and conclusions

While there is no doubt about the importance of insurance on economic growth, insurance penetration in Africa remains the lowest globally. To increase the impact of insurance penetration on economic growth, the African Insurance Organisation emphasises the need for structural transformation. This has become essential given that African countries are rethinking their structural transformation strategies and exploring new change patterns following the 2030 Sustainable Development Agenda, which envisages higher structural transformation. However, this is yet to be empirically tested. Against this backdrop, this study contributes to the debate by exploring how insurance could be developed in Africa. For the first time in the literature, this study provides initial evidence of how structural transformation moderates the relationship between insurance and growth, which, to the best of our knowledge, is yet to be rigorously examined. It further explores nonlinearities in the relationship to determine whether the impact differs across different levels. A two-step system GMM and dynamic panel threshold estimation techniques were used for analysis, spanning 2008–2020, based on thirty-eight (38) African countries.

The baseline result, which explores the direct impact, reveals a positive relationship between insurance and economic growth, which shows that insurance is essential to the growth of economies. Notwithstanding, the result was statistically insignificant, implying that the current level of insurance is not strong enough to drive growth. Further, the structural transformation index presents a significant positive impact on economic growth, suggesting that improving the structural reforms will accelerate economic growth. Similarly, all the individual factors, except agriculture, positively induce economic growth. This indicates the importance of structural transformation in driving the economic performance of African economies. Hence, there is a need to promote structural change, including technological and sectoral development and urbanisation, to drive economic growth. Regarding the interaction between insurance and structural transformation, the study finds that, except for infant mortality, all the structural transformation variables positively interact with insurance in affecting economic growth. Again, the interaction between the structural transformation index and insurance penetration is significantly positive. This is an indication that high structural transformation is likely to propel the impact of insurance on economic growth. Additionally, the results from the threshold analysis document nonlinearities in the relationship between insurance and economic growth in the form of a U-shape. It further presents a significant threshold of 2.12. Beyond the threshold level, insurance exhibits a positive relationship with economic growth and vice versa. This shows that growth in the insurance market is imperative for economic growth. In addition, the study finds that the interaction between insurance and structural transformation reveals a similar pattern, indicating a significant positive effect in the upper regime and a negative impact in the lower regime. This suggests the need for strong structural reforms in Africa because high structural transformation propels the impact of insurance on economic growth in Africa. In other words, the development of the insurance market is driven by the structural environment. It also indicates that structural transformation policies are crucial because they improve insurance penetration in Africa, ultimately affecting economic growth.

### 5.2 Theoretical implications

The positive association between insurance and economic growth supports the supply-leading hypothesis, which establishes that financial services such as those provided by the insurance industry stimulate economic growth by offering policies and risk management services. This ensures stability and growth in business, which promotes economic performance. Further, the theory stipulates that insurance encourages innovation, investment and overall economic activity. This greatly resonates with the theory of financial intermediation, which highlights the important role of financial institutions towards economic growth. This theory posits that financial institutions, including the insurance industry, promote economic activity by offering capital mobilisation, risk management and investment services. This underscores the crucial role of the insurance industry towards the economy. Hence, improving the effectiveness and accessibility of insurance services can bolster their contribution to business stability and economic growth. Additionally, the moderating influence of structural transformation on the insurance-growth nexus affirms the institutional and economic transformation theories. According to these theories, structural transformation, which involves changes from traditional or low productivity to more diverse economic activities and higher productivity, creates new institutional environments for the development of the insurance industry. For example, transitioning from an agricultural-dependent economy to an industrial and service-oriented environment increases the demand for new and diversified insurance products, improving insurance capacity and driving economic growth. More so, as economies move towards industrialisation, urbanisation and technological development, the risk and demands for insurance increase, which provides an avenue for insurance market development, strengthening their role in fostering economic growth.

### 5.3 Policy implications

The findings of this study present several policy implications. Given the positive relationship between insurance and economic growth, the study suggests that policymakers should prioritise the development of the insurance industry by creating comprehensive strategies for growth. This includes creating an enabling environment for insurance growth, bolstering their capacity to pool risks, and enhancing customer service. This will likely lead to greater resilience and financial stability, which are necessary to sustain long-term economic growth. More so, targeted policies that promote the purchase of insurance products, such as tax incentives, can also improve the expansion of the industry. This is very important to capture the mass population, given the lack of confidence of the populace in most parts of the region. Furthermore, insurance companies could use conferences, seminars and advertisements to increase public awareness of their products. They should also implement several initiatives aimed at educating customers on the benefits of insurance to promote insurance trust. This will correct most people’s wrong perception of insurance as an unnecessary expenditure. Additionally, the limited insurance product lines in the African market today do not adequately handle the variety of risks individuals and companies face. Hence, efforts to introduce new insurance products, including small business and index-based insurance, are required to boost the insurance market. This also requires efficient and effective risk pricing strategies and statistical tools for effective underwriting.

Further, given the substantial impact of insurance above the threshold level, the study suggests that higher insurance penetration is essential to economic growth. In light of this, policy discussions must align with the estimated threshold level, thus at least 2 %. Thus, insurance industries in Africa should strive to reach the minimum threshold level. Given that most African countries fall below the threshold level, governments and regulatory authorities must concentrate on strategies for advancing the insurance market to achieve this critical mass. This requires tailoring insurance products to the needs and demands of Africa, particularly those in rural areas. Given that a large portion of the African population resides in rural areas and is the most vulnerable, targeting this population and developing affordable insurance policies to address the needs of rural communities and urban-poor individuals presents significant opportunities to develop the insurance market, thereby enhancing the growth of economies. This may include microinsurance, agricultural insurance and health and index-based insurance, which are required to boost the insurance market. Such developments in the insurance market will ultimately drive economic growth.

It is important to note that insurance alone is not enough to boost economic growth, but with the presence of a strong structural transformation, the overall benefits of insurance will be realised. Thus, the insurance market will thrive within an environment with strong structural reforms. Given this, there is a need for government intervention and policies to improve the structural reforms in African countries. This also requires the integration of the insurance industry policies with the overall economic transformation agenda. Moreover, the insurance industry must acknowledge the importance of structural transformation to the insurance market and develop appropriate policies to speed up access to the underinsured and increase insurance penetration. Policies that strengthen the ties between insurance and other sectors, including the industrial, manufacturing and service sectors, must be considered. As African economies work toward industrialisation, policies may mandate or promote insurance coverage for new investments in the manufacturing and industrial sectors. This requires incorporating insurance policies into industrial policy, such as business interruption insurance, compulsory fire insurance, and public-private partnership agreements. Businesses in these sectors would be protected from risks and would promote stability in their growth. These interventions will provide new opportunities to the insurance market and promote economic growth. More so, the service sector could benefit from professional indemnity and liability insurance.

In addition, given the rapid urbanisation in Africa, insurers could leverage this opportunity to develop targeted products for houses, public infrastructure, health and disaster management and businesses to sustain economic activities. By integrating insurance policies with the various sectors, the market will be developed, ultimately translating into economic growth. Similarly for ICT, governments can contribute by providing the resources for developing ICT infrastructure due to its importance in driving the insurance market in this digital era. Moreover, the insurance industry should consider partnerships with technology companies to help digitise their products and services. This will help attract more customers online, beyond their geographical jurisdiction, driving insurance development. More so, insurers can leverage new technologies to develop digital policies for insurance products. This will overcome the traditional barriers to insurance business and increase public awareness, thereby enhancing public trust and penetration levels. This will ultimately accelerate the development of the insurance market and economic growth. In summary, the study suggests that African countries with low structural transformation should develop policies to enhance their structural reforms. This has huge potential to leapfrog the insurance market, ensuring its development, which will, in turn, affect economic growth.

### 5.4 Limitations and future research directions

Although this study offers insightful information, there are a few limitations that should be taken into account for future research. A major limitation of this study is that it relies on panel data from several African countries. While this provides an interesting perspective from the African context, country-specific analysis may be required to understand how specific structural and contextual factors might influence the relationship. Hence, country-level and regional case studies are recommended. The study’s inability to estimate multiple threshold levels using the dynamic panel threshold technique is another major drawback; thus, future research could consider this as a way to capture the underlying intricacies in the model. More so, the evidence from this study provides a call for comparative analysis by exploring the moderating role of structural transformation on the insurance-growth nexus from developed and other emerging markets. This will provide insight into the pace of structural transformation and how it affects the insurance market. It is also important to re-examine the impact of structural transformation on the life and non-life insurance industry in Africa due to the difference in business operations. Given the substantial potential for growth of the insurance business, additional studies may consider other moderating factors, such as human capital development and financial inclusion in the relationship.

## CRediT authorship contribution statement

**Sylvester Senyo Horvey:** Writing – review & editing, Writing – original draft, Visualization, Validation, Software, Methodology, Formal analysis, Data curation, Conceptualization. **Jones Odei-Mensah:** Writing – review & editing, Writing – original draft, Validation, Supervision. **Andre P. Liebenberg:** Writing – review & editing, Writing – original draft, Validation, Supervision.

## Data availability statement

Data will be made available on request.

## Declaration of competing interest

The authors declare that they have no known competing financial interests or personal relationships that could have appeared to influence the work reported in this paper.

## Figures and Tables

**Table 6:** Direct effects of insurance and structural transformation on economic growth (Dep Var: GDP per capita).

	(1)	(2)	(3)	(4)	(5)	(6)	(7)	(8)	(9)
Lag of GDP	0.278∗∗∗	0.268∗∗∗	0.273∗∗∗	0.276∗∗∗	0.272∗∗∗	0.277∗∗∗	0.299∗∗∗	0.290∗∗∗	0.268∗∗∗
	(0.090)	(0.087)	(0.087)	(0.086)	(0.091)	(0.073)	(0.064)	(0.080)	(0.087)
FDI	−0.432	−0.411	−0.535∗∗	−0.432	−0.442	−0.352	−0.295	−0.264	−0.471
	(0.294)	(0.287)	(0.246)	(0.288)	(0.306)	(0.223)	(0.200)	(0.236)	(0.387)
Remittances	−0.819∗	−0.859∗	−0.813∗∗	−0.851∗	−0.843∗	−0.658	−0.335	−0.433	−0.859∗
	(0.462)	(0.451)	(0.397)	(0.420)	(0.477)	(0.493)	(0.334)	(0.332)	(0.451)
Trade	0.088	0.087	0.142∗∗	0.097∗	0.089	0.075	0.029	0.031	0.087
	(0.066)	(0.064)	(0.058)	(0.055)	(0.068)	(0.056)	(0.046)	(0.051)	(0.054)
Inflation	−0.015∗∗∗	−0.016∗∗∗	−0.012∗∗∗	−0.015∗∗	−0.016∗∗	−0.012∗∗	−0.008∗∗	−0.009∗	−0.016∗∗∗
	(0.006)	(0.006)	(0.004)	(0.006)	(0.006)	(0.005)	(0.004)	(0.005)	(0.006)
Insurance	0.022								
	(0.032)								
Industry		7.297							
		(6.073)							
Agriculture			−3.615∗∗						
			(1.124)						
Manufacturing				−4.834					
				(4.453)					
Service					3.665				
					(7.670)				
Urbanisation						0.051			
						(0.104)			
ICT							0.094∗∗∗		
							(0.030)		
Mortality								0.164∗∗∗	
								(0.054)	
Structural Transformation									1.348∗∗
									(0.444)
Year and Country Dummies	Yes	Yes	Yes	Yes	Yes	Yes	Yes	Yes	Yes
Observations	455	455	455	455	455	455	455	455	455
Number of groups	38	38	38	38	38	38	38	38	38
Number of instruments	11	11	11	11	11	11	11	11	11
Prob > F	0.000	0.000	0.000	0.000	0.000	0.000	0.000	0.000	0.000
AR(1)	0.000	0.000	0.001	0.000	0.000	0.000	0.000	0.000	0.000
AR(2)	0.274	0.318	0.111	0.332	0.265	0.251	0.290	0.391	0.318
Hansen	0.121	0.095	0.092	0.088	0.115	0.049	0.429	0.433	0.095

Note: Standard errors in parentheses; Significant levels are expressed as ∗∗∗p < 0.01, ∗∗p < 0.05, ∗p < 0.1, respectively.

**Table 7:** The interaction effect of insurance and structural transformation on economic growth (Dep Var: GDP per capita).

Variables	(10)	(11)	(12)	(13)	(14)	(15)	(16)	(17)
Lag of GDP	0.282∗∗∗	0.325∗∗	0.263∗∗∗	0.285∗∗∗	0.292∗∗∗	0.290∗∗∗	0.273∗∗∗	0.282∗∗∗
	(0.082)	(0.145)	(0.084)	(0.085)	(0.083)	(0.073)	(0.089)	(0.082)
Insurance	0.012	−0.408	0.067	0.034	−0.510	0.088	5.356	2.436∗∗
	(0.029)	(0.342)	(0.074)	(0.040)	(0.844)	(0.065)	(4.804)	(1.174)
FDI	−0.476∗	−0.686	−0.454	−0.479	−0.401	−0.314	−0.318	−0.476∗
	(0.269)	(0.520)	(0.290)	(0.326)	(0.256)	(0.278)	(0.300)	(0.269)
Remittances	−0.831∗	−0.887	−0.826∗	−0.840∗	−0.724∗	−0.354	−0.497	−0.831∗
	(0.419)	(0.703)	(0.450)	(0.490)	(0.421)	(0.418)	(0.405)	(0.419)
Trade	0.091∗	0.145∗	0.095∗	0.081∗	0.072∗	0.032	0.046	0.091∗
	(0.051)	(0.077)	(0.056)	(0.059)	(0.048)	(0.060)	(0.061)	(0.051)
Inflation	−0.016∗∗∗	−0.015∗∗	−0.015∗∗	−0.017∗∗	−0.013∗∗	−0.009	−0.009	−0.016∗∗∗
	(0.005)	(0.007)	(0.006)	(0.006)	(0.006)	(0.007)	(0.007)	(0.005)
Industry	1.086							
	(2.312)							
Insurance∗Industry	3.457∗∗							
	(1.655)							
Agriculture		−6.076∗						
		(3.241)						
Insurance∗Agriculture		7.006∗						
		(4.240)						
Manufacturing			−5.398					
			(4.222)					
Insurance∗Manufacturing			0.532					
			(0.538)					
Service				3.411				
				(5.355)				
Insurance∗Service				0.581				
				(1.202)				
Urbanisation					−0.048			
					(0.113)			
Insurance∗Urbanisation					0.016			
					(0.025)			
ICT						−0.162∗∗∗		
						(0.053)		
Insurance∗ICT						0.066		
						(0.049)		
Mortality							0.260∗∗	
							(0.117)	
Insurance∗Mortality							−0.073	
							(0.065)	
STI								1.689∗∗∗
								(0.071)
Insurance∗STI								3.376∗∗
								(1.474)
Net Effects	0.033	0.046	0.074	0.039	0.154	1.234	1.713	2.903∗
Year and Country Dummies	Yes	Yes	Yes	Yes	Yes	Yes	Yes	Yes
Observations	455	455	455	455	455	455	455	455
Number of groups	38	38	38	38	38	38	38	38
Number of instruments	13	13	13	13	13	13	13	13
Prob > F	0.000	0.000	0.000	0.000	0.000	0.000	0.000	0.000
AR(1)	0.000	0.006	0.000	0.000	0.000	0.000	0.000	0.000
AR(2)	0.335	0.218	0.487	0.296	0.260	0.484	0.475	0.335
Hansen p-value	0.111	0.268	0.122	0.145	0.074	0.512	0.444	0.111

**Note:** Standard errors are in parentheses; Significant levels are expressed as ∗∗∗p < 0.01, ∗∗p < 0.05 and ∗ p < 0.1, respectively. Where GDP represents economic growth; FDI represents foreign direct investment; ICT represents information and communication technology; STI represents the overall structural transformation index. **Source:** Results were generated from Stata by authors.

**Table 8:** Threshold model with interaction variable (Dep Var: GDP per capita).

VARIABLES	Lower Regime		Upper Regime
Lag of GDP	0.517∗∗		0.142∗∗
	(0.287)		(0.029)
Insurance	−1.765∗		1.346∗
	(1.034)		(0.887)
Insurance∗STI	−1.563∗∗		2.043∗∗
	(0.764)		(0.908)
STI	2.762∗		3.123∗∗
	(1.371)		(1.105)
FDI	−0.235∗∗		0.428∗∗
	(0.076)		(0.189)
Remittances	−0.676		1.653
	(0.547)		(1.132)
Trade	0.034		0.778∗∗∗
	(0.020)		(0.146)
Inflation	−0.002∗∗		0.005∗∗
	(0.001)		(0.002)
**Post-estimations**			
Threshold Value		1.945∗∗∗	
		(0.081)	
Linearity Test (P-value)		0.000	
Observations		38	

**Note:** Standard errors are in parentheses; Significant levels are expressed as ∗∗∗p < 0.01, ∗∗p < 0.05 and ∗ p < 0.1, respectively. Where GDP represents economic growth; FDI represents foreign direct investment; and STI represents the overall structural transformation index. **Source:** Results were generated from Stata by authors.

## Footnotes

1 The list of countries includes Angola, Benin, Botswana, Burkina Faso, Burundi, Cabo Verde, Cameroon, Chad, DR Congo, Congo Republic, Côte d’Ivoire, Equatorial Guinea, Eswatini, Ethiopia, Gabon, The Gambia, Ghana, Guinea, Kenya, Lesotho, Madagascar, Mali, Mauritania, Mauritius, Mozambique, Namibia, Niger, Nigeria, Rwanda, Senegal, Seychelles, Sierra Leone, South Africa, Tanzania, Togo, Uganda, Zambia and Zimbabwe.

2 We check the correlations between the seven variables, identified and retained the subset of factors that explains the most variance in the underlying variables using the eigenvalues. Three factors were extracted because their eigenvalues were closer to or beyond 1. Factor 1 has eigenvalue of 2.26 and explains 43 % of the overall variance. Factor 2 records an eigenvalue of 1.41 and explains 24 % variance while factor 3 has an eigenvalue of 1.13 and accounts for 15 %. Generally, these factors explain 82 % of the overall variance. Individual factors with high loading factors were minimised using the orthogonal varimax rotation (kaiser off) to determine the contribution of each factor, we then find the total of each underlying variable’s contribution to all retained factors to derive the structural transformation index.
